# Enrofloxacin and Sulfamethoxazole Sorption on Carbonized Leonardite: Kinetics, Isotherms, Influential Effects, and Antibacterial Activity toward *S. aureus* ATCC 25923

**DOI:** 10.3390/antibiotics11091261

**Published:** 2022-09-16

**Authors:** Chanat Chokejaroenrat, Chainarong Sakulthaew, Khomson Satchasataporn, Daniel D. Snow, Tarik E. Ali, Mohammed A. Assiri, Apichon Watcharenwong, Saksit Imman, Nopparat Suriyachai, Torpong Kreetachat

**Affiliations:** 1 Department of Environmental Technology and Management, Faculty of Environment, Kasetsart University, Bangkok 10900, Thailand; 2 Department of Veterinary Technology, Faculty of Veterinary Technology, Kasetsart University, Bangkok 10900, Thailand; 3 Water Sciences Laboratory, Nebraska Water Center/School of Natural Resources, University of Nebraska—Lincoln, Lincoln, NE 68583-0844, USA; 4 Department of Chemistry, Faculty of Science, King Khalid University, Abha 62529, Saudi Arabia; 5 Department of Chemistry, Faculty of Education, Ain Shams University, Cairo 11566, Egypt; 6 School of Environmental Engineering, Institute of Engineering, Suranaree University of Technology, Nakhon Ratchasima 30000, Thailand; 7 Center of Excellence in Advanced Functional Materials, Suranaree University of Technology, Nakhon Ratchasima 30000, Thailand; 8 Integrated Biorefinery Excellent Center (IBC), School of Energy and Environment, University of Phayao, Tambon Maeka, Amphur Muang, Phayao 56000, Thailand

**Keywords:** adsorption isotherm, adsorption kinetics, antibiotic adsorption, carbonization, elovich, enrofloxacin, growth inhibition zone, intraparticle diffusion, leonardite, sulfamethoxazole

## Abstract

Excessive antibiotic use in veterinary applications has resulted in water contamination and potentially poses a serious threat to aquatic environments and human health. The objective of the current study was to quantify carbonized leonardite (cLND) adsorption capabilities to remove sulfamethoxazole (SMX)- and enrofloxacin (ENR)-contaminated water and to determine the microbial activity of ENR residuals on cLND following adsorption. The cLND samples prepared at 450 °C and 850 °C (cLND450 and cLND550, respectively) were evaluated for structural and physical characteristics and adsorption capabilities based on adsorption kinetics and isotherm studies. The low pyrolysis temperature of cLND resulted in a heterogeneous surface that was abundant in both hydrophobic and hydrophilic functional groups. SMX and ENR adsorption were best described using a pseudo-second-order rate expression. The SMX and ENR adsorption equilibrium data on cLND450 and cLND550 revealed their better compliance with a Langmuir isotherm than with four other models based on 2.3-fold higher values of q_mENR_ than q_mSMX_. Under the presence of the environmental interference, the electrostatic interaction was the main contributing factor to the adsorption capability. Microbial activity experiments based on the growth of *Staphylococcus aureus* ATCC 25923 revealed that cLND could successfully adsorb and subsequently retain the adsorbed antibiotic on the cLND surface. This study demonstrated the potential of cLND550 as a suitable low-cost adsorbent for the highly efficient removal of antibiotics from water.

## 1. Introduction

An exponential increase in medicinal technology development has resulted in excessive antibiotic use in veterinary practices. In many countries, farmers use antibiotics not only therapeutically but also prophylactically (as growth promoters) in livestock, poultry farming, and aquaculture in an effort to gain a substantial additional yield in a relatively shorter time frame [1,2,3]. The excess antibiotic may enter the environment via animal waste excretion or in discharge water and may even exist in active forms, as it can be partially metabolized by animals [4,5,6,7]. These residuals (estimated at 30–90% of the initial load) are mostly still considered to be pharmaceutically active compounds. Without proper waste and discharge water treatment, these chemicals can potentially contaminate soils, surface water, and ground water and pose a serious threat to the environment [8,9,10,11,12]. Long-term exposure to these contaminated antibiotics is considered to be directly interrelated to the proliferation of antibiotic resistance genes, which can be genetically transferred among local microorganisms [13,14].

The sulfonamide, macrolide, and tetracycline antibiotic families have been mostly detected at microgram-per-liter levels in tropical Asian countries [15]. Among these chemicals, sulfamethoxazole (SMX) has been detected at up to 1720 ng L^−1^, which is higher than the detected levels in China and Western countries. Antibiotic residuals in conventional wastewater treatment plants (WWTPs) have been regularly reported throughout the world. The fluoroquinolone and sulfonamide families have been frequently detected in WWTPs and natural receiving water, such as canals, and rivers in Thailand [16]. Following chlorination and UV irradiation, SMX has proven to be the most difficult antibiotic to remove among the sulfonamides [17]. Up to 21 μg L^−1^ of ampicillin was detected in the effluent water from an Indian city municipal WWTP [18]. Even with the strict regulation of drug manufacturers in India, up to 31,000 μg L^−1^ of ciprofloxacin—from enrofloxacin degradation—was discovered in the effluent [19]. This evidence signifies the potential inadequacy of regular, conventional unit operations, such as chlorination, biological treatment, filtration, and coagulation. Hydroxyl radical (^•^OH) generation in advanced oxidation processes provides an efficient approach for mitigating antibiotics [20]. However, this treatment usually requires a large amount of oxidant and has high operational and application costs, as well as possibly leading to secondary contamination [21,22].

Adsorption was proven to be an efficient treatment meeting both economic values and environmental requirements. Although carbon nanomaterial is usually applied as a decent antibiotics adsorbent, these materials are not economical for large-scale treatments [9,23,24]. To overcome this problem, pyrolyzed material is preferentially selected, as it generally enriches with carbon, which is naturally suitable for removing organic contaminants. The pyrolyzed materials tend to possess a more porous structure, high surface area, and high cation exchange capacity that are more compatible with antibiotics capable of presenting in different ionic forms. The magnitude of antibiotic sorption is based on various mechanisms such as the hydrophobic interaction, π–π interaction, H bonding, and even electrostatic interaction [25,26]. Example of pyrolyzed material that efficiently adsorb antibiotics included animal manure, rice straw, etc. [27,28].

Uncarbonized leonardite (LND)—a byproduct from a lignite coal reaction in a power plant—is naturally abundant in humic substances, consisting of a wide variety of both carboxylic and hydroxylic sites, making it a promising material as a soil conditioner and an extremely porous material after pyrolysis (as carbonized LND or cLND). Recently, leonardite char (cLND) was used as a suitable adsorbent for both organic and inorganic pollutants, such as atrazine, organic dyes, and heavy metals [29,30,31]. The cLND not only had abundant C=C structures on the surface but also had numerous fractures on its planar surface, making it very suitable for adsorbing both hydrophobic and hydrophilic materials [31]. While cLND chemical and physical properties are well-understood, only limited information has been reported on cLND adsorptive application with emerging contaminants such as antibiotics.

In this study, we selected two frequently used veterinary antibiotics—sulfamethoxazole (SMX) and enrofloxacin (ENR)—as adsorbate antibiotic representatives, because they were the most frequently found antibiotics in aquacultural discharge wastewater and natural flowing streams [32]. The objectives were: (1) to quantify the ENR and SMX removal efficiencies using cLND based on adsorption kinetics and adsorption isotherms under different experimental conditions and (2) to determine the microbial activities on the adsorbed ENR on cLND using *Staphylococcus aureus* ATCC 25923 during the adsorption and desorption processes.

## 2. Material and Methods

### 2.1. Chemicals and Chemical Analyses

The representative antibiotics used in this study—enrofloxacin (ENR) and sulfamethoxazole (SMX)—were purchased from Merck. Sodium hydroxide, hydrochloric acid, sodium bicarbonate, acetic acid, and potassium chloride were used as purchased from Carlo Erba. Acetonitrile and methanol were of high-performance liquid chromatography (HPLC) grade obtained from Honeywell Burdick & Jackson. Low-grade coal (leonardite; LND) was donated from the Mae Moh lignite mine in Lampang Province, Thailand.

The antibiotics were analyzed using HPLC in a 600E unit coupled with a 2487 UV detector (Waters). The mobile phase used for the ENR analysis was a mixture of 0.1% acetic acid (*v*/*v*) and acetonitrile at 80:20, while that used for the SMX analysis was at 60:40. Peak separation was achieved using a Mightysil HPLC column (RP-18GP; 250 × Ø4.6 mm, particle size 5 μm, pore size 12.5 nm; Kanto Chemical) under a flow rate of 1 mL min^−1^ at room temperature. The injection volume was set at 20 μL, and the detection wavelengths were set at 265 and 280 nm for SMX and ENR, respectively.

We compared the cLND physical and chemical characteristics at different carbonized temperatures. The cLND morphological properties were obtained from scanning electron microscopy (SEM; JEOL JSM-6010). The surface functional groups were analyzed using Fourier-transform infrared spectroscopy (FTIR; Bruker Tensor 27).

### 2.2. cLND Preparation

Initially, we discarded distinctively large and darkened debris from the obtained LND before drying the remainder in a hot air oven at 105 °C for 3 h. Since the LND varied in size, we mechanically sieved using an AS200 sieve shaker (Retsch GmbH) and sorted only the <2 nm particles. Then, the LND was carbonized at a 5 °C min^−1^ heat rate to the desired carbonized temperatures of 450 °C, 550 °C, 650 °C, 750 °C, and 850 °C (denoted as cLND450 for the 450 °C carbonizations). To maintain the cLND pyrolysis conditions, the heating system was maintained for 5 h at each designated temperature under an N_2_ flow stream. The cLND powder was kept in a desiccator at room temperature until use.

### 2.3. Adsorption Kinetics

The ENR and SMX stock solution was freshly prepared before use to ensure its stability at room temperature. A sample of 30 mL of 100 mg L^−1^ antibiotic solution was placed individually in a 40-mL amber vial with a Teflon-lined screw cap. An experimental unit was done in triplicate. Each vial received 30 mg cLND and was shaken on a reciprocating shaker at 200 rpm. The cLND carbonized at varying temperatures (i.e., 450, 550, 650, 750, and 850 °C) was tested individually. Samples were periodically collected, placed in separate 1.5-mL centrifuge tubes, centrifuged at 5000 rpm for 10 min, transferred to an HPLC vial, and stored at 4 °C before analysis using HPLC.

### 2.4. Adsorption Isotherm

The isotherm experiments were conducted in a 40-mL Teflon tube. Only cLND450 and cLND550 were selected as an absorbent for both ENR and SMX. Each antibiotic solution of 30 mL with an initial concentration ranging between 5 and 50 mg L^−1^ was separately poured into each vial. The isotherm experiment commenced when the pre-weighed cLND at 30 ± 0.2 mg was added to each vial. The cLND exact weights were recorded for further adsorbed concentration calculations. The sample collection protocol was similar to the previous experiment. However, we only collected samples twice (at 24 h and 48 h) to confirm that an equilibrium between the cLND and antibiotic had been reached. Then, the adsorbed concentration ($q_{e}$) and equilibrium concentration ($C_{e}$) were plotted and determined for the best fit adsorption isotherms.

### 2.5. Point of Zero Charges Determination

Since the point of zero charge (pH_zpc_) can explain the material adsorption capacity under the presence of ionic constituents, we determined the pH_zpc_ using changes in the pH before and after adding cLND. Eleven flasks containing 50 mL of sodium chloride solution (0.01 M) were prepared. Each flask was pH-adjusted to designate pH (2–12) using 0.1 M of either HCl or NaOH. A small amount (0.12 g) of cLND was placed in each flask, and the pH was immediately measured. The first pH curve was plotted between the before and after adjusted pH. Then, all flasks were agitated at 180 rpm for 48 h. The final pH was recorded and plotted against the initial pH. The pH_zpc_ can be obtained from the intersection of both pH curves. 

### 2.6. Influential Effect Experiments

Since other environmental parameters are known to directly affect the adsorbent surface charge, we individually varied the parameters such as solution pH, humic acid (HA), HCO_3_^−^, and Cl^−^ and measured the temporal changes in the antibiotic concentrations. The ranged concentrations of these values were based on the normal values that could be observed under natural conditions. The solution pH ranged from 3 to 11, the humic acid concentrations ranged from 2.5 to 40 mg L^−1^, and the HCO_3_^−^ and Cl^−^ concentrations ranged from 50 and 800 mg L^−1^.

### 2.7. ENR Bacterial ACTIVITY after Treatment

Six ENR concentrations ranging between 10 and 100 mg L^−1^ were tested in this experiment to differentiate the cLND adsorption and desorption activities from the adsorption treatments between two cLNDs (cLND450 and cLND550) at the two cLND amounts (30 and 300 mg). The experimental setup was arranged similarly to the adsorption kinetic experiments discussed earlier. We investigated the bacterial activities of cLND-adsorbed ENR against Gram-negative *Staphylococcus aureus* (ATCC 25923) bacteria using the agar diffusion technique based on the Kirby–Bauer method. In brief, colonies of bacteria-grown culture were adjusted to an opacity equivalent to 0.5 McFarland (~1.5 × 10^8^ CFU mL^−1^), seeded on Mueller–Hinton agar in a petri dish, and grown overnight under aerobic conditions at 37 °C.

After the cLND-ENR reaction reached an adsorption equilibrium at 24 h, two separate samples were taken from the adsorption experimental units: (1) filtrated water and (2) used cLND (the retentate). We passed the filtrated watered using a vacuum filter through a 0.22-μm PES Corning filter membrane (Glendale, AZ, USA). Ten microliters of filtrated sample were dropped directly on a sterile blank disc (Ø = 6 mm, Whatman, USA), dried in the dark for 30 min, and placed on the bacterial inoculated agar. A standard ENR antibiotic disc (5 µg; Oxoid, UK) was used as the positive control. The culture plates were further incubated at 37 °C for 24 h. At the designated time following incubation, a symmetrical inhibition ellipse was measured using a reflected lightbox and digital calipers. To evaluate how cLND strongly adsorb ENR, we gently placed a sterile blank disc over the retentate cLNDs and dried them in the dark for 30 min. Each disc was placed in a separate bacterial inoculated agar and incubated at 37 °C for 24 h. Then, the increase in inhibition zones was compared between day 1 and day 3.

## 3. Results and Discussion

### 3.1. cLND Characteristics

**Figure 1:**
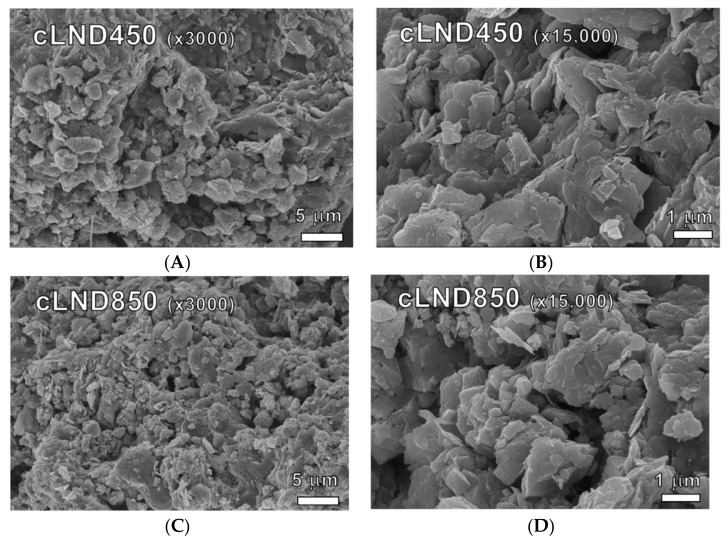
(**A**–**D**) cLND scanning electron micrograph images at 450 °C and 850 °C carbonization temperature (cLND450 and cLND850) at 3000 magnification and 15,000 magnification.

A carbonized LND (cLND) morphological analysis was performed using SEM and FTIR. The cLND SEM images of two carbonization temperatures at 450 °C and 850 °C (cLND450 and cLND850) were viewed at 3000 and 15,000 magnification (Figure 1). These images indicated clear heterogeneity crowded with irregular lamella-shaped flakes overlaying each other. While irregular surface pores were observed in both carbonization temperatures, those in the cLND850 were more evident, indicating that the molecular pore-filling mechanism may be prominent at higher carbonization temperatures (Figure 1C,D). This was the initial proof that the cLND was a suitable adsorbent for both organic and inorganic compounds. High carbonization temperatures at > 550 °C eliminated the organic compounds (the carboxylic group), resulting in a rougher surface, greater hydrophobicity and porosity, and a large surface area, which provided a greater surface area and consequently provided more binding sites with adsorbate, making it a docile adsorbent, particularly with organic pollutants [29,30,31,33]. With lower carbonization temperatures (< 550 °C), the biochar typically had smaller pore sizes with oxygen-containing functional group residuals on the surface, rendering them well-suited for inorganic pollutant adsorption [30]. Nonetheless, the higher pyrolysis temperature did not necessarily ensure better adsorption, and this was also the case for cLND.

**Figure 2:**
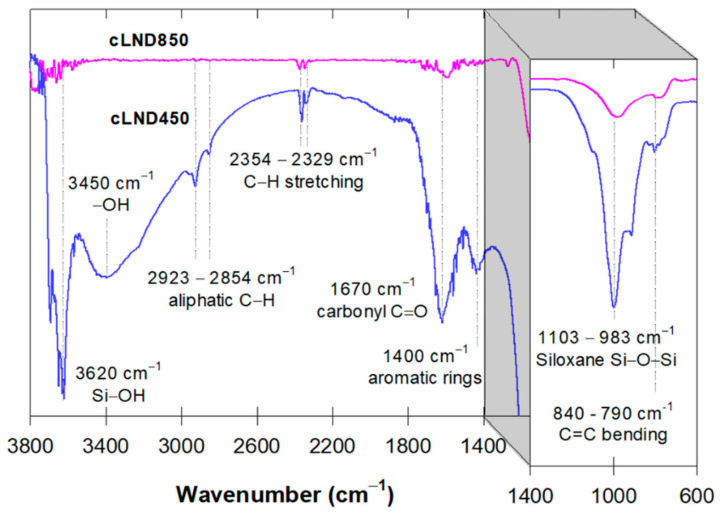
FTIR spectra comparisons between cLND450 and cLND850.

The cLND FTIR spectra were recorded in the range 600–3800 cm^−1^ to characterize the surface functional group changes between the two carbonization temperatures. Some of these changes reflected the cLND adsorptive effectiveness toward organic molecules. The higher carbonization temperature caused the loss of a stronger and broader band at 3200–3600 cm^−1^, which was associated with the stretching vibration of the –OH groups (Figure 2). These changes in the functional groups initially confirmed that the cLND adsorption behavior could be categorized as both hydrophobic and hydrophilic interactions. The cLND450 FTIR spectra showed stronger bands of symmetrical and asymmetrical aromatic and aliphatic C-H bonding (CH_3_-CH_2_) at 2923–2854 cm^−1^, while these bands almost disappeared during higher carbonization (Figure 2). A strong adsorption band at 3450 cm^−1^, corresponding to the –OH groups, was more evident at the lower carbonization temperature similar to other hydrophilic functional groups, such as H-bonded OH, C = O, and Si-O-Si located at 3620, 1670, and 1100 cm^−1^, respectively (Figure 2). The peak at 3620 cm^−1^ could be attributed to the H-bonded OH of the Si-OH group or other H bonding with water molecules that almost completely dissipated at the higher carbonization temperature. These results indicated that cLND850 was less polar than cLND450, supporting the beneficial use of cLND450 for hydrophilic/hydrophobic interactions and cLND850 for hydrophobic interactions.

### 3.2. Adsorption Kinetics

**Figure 3:**
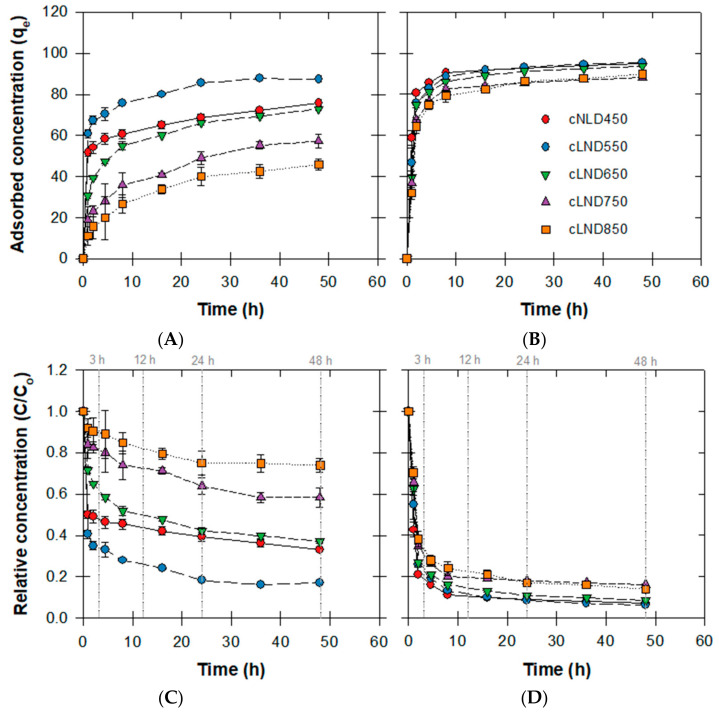
Adsorption kinetic studies of varying cLND with SMX or ENR. (**A**,**B**) Temporal changes in the antibiotic concentrations. (**C**,**D**) Temporal changes in the adsorbed concentrations in cLND.

In the kinetic experiments of both antibiotics and each cLND at different carbonization temperatures (450–850 °C), both the adsorbed concentration ($q_{e}$) and relative adsorption concentration (C/C_o_) were plotted against time (Figure 3). For cLND that carbonized at 450–650 °C, a rapid decrease in antibiotic adsorption was observed (SMX < 3 h, ENR < 1 h), as shown in Figure 3C,D, respectively. At the higher carbonization temperature, slower SMX adsorption rates were observed, while the ENR adsorption rates were slightly different. Although the increase in contact time increased the adsorption performance, it mainly depended on the cLND type for SMX adsorption (Figure 3A,C). Each SMX adsorption result tended to reach a plateau at a specific adsorbed amount, indicating that the carbonization temperature directly affected the SMX adsorption performance. The ENR adsorption was substantially different compared to that of SMX (Figure 3B,D). All of them reached a plateau at relatively close $q_{e}$ values, supporting the adsorbate molecular structure as the main contributing factor for better adsorption. However, a larger cLND amount tended to agglomerate into a bulkier size, decreasing the adsorptive sites in the cLND microspores [31]. Here, we did not observe such a case, as there was not enough cLND to initiate such an agglomeration. Overall, the results initially proved that cLNDs were an efficient adsorbent for both antibiotics, but the SMX and ENR adsorption rates were substantially different due to (1) the SMX and ENR chemical perspectives and (2) the differences in the carbonization temperatures. 

We used four kinetic models to explain the adsorption process between each antibiotic and cLND at varying temperatures (450–850 °C). These four models were: pseudo-first-order kinetics, pseudo-second-order kinetics, Elovich, and intraparticle diffusion (Equations (1)–(4), respectively) [34].

(1) $$\mathrm{Pseudo}-\mathrm{first}-\mathrm{order}\;\mathrm{kinetic}\;\mathrm{model}:\;q_{t}=q_{e}\left(1-e^{-K_{1}t}\right)\;\mathrm{or}\;(q_{e}-q_{t})=q_{e}e^{-K_{1}t}$$

(2) $$\mathrm{Pseudo}-\sec\mathrm{ond}-\mathrm{order}\;\mathrm{kinetic}\;\mathrm{model}:\;\frac{t}{q_{t}}=\frac{1}{K_{2}q_{e}^{2}}+\frac{1}{q_{e}}t$$

(3) $$\mathrm{Elovich}\;\mathrm{model}:\;q_{t}=\left(\frac{1}{b}\right)\ln\left(ab\right)+\left(\frac{1}{b}\right)\ln t$$

(4) Intraparticle diffusion: qt=Kdt12+C

where $q_{e}$ and $q_{t}$ (mg g^−1^) are the cLND adsorption capacity at equilibrium and at any given time t (h), respectively, $K_{1}$ (h^−1^) is the rate constant of the pseudo-first-order model, $K_{2}$ (g mg^−1^h^−1^) is the rate constant of the pseudo-second-order model, a (mg g^−1^h^−1^) is the Elovich chemisorption rate, b (g mg^−1^) is the desorption rate constant, $K_{d}$ (mg g^−1^h^−1/2^) is the intraparticle diffusion coefficient, and C (mg g^−1^) is the intraparticle diffusion constant.

Two correlation values were selected to evaluate the validity of each model: the coefficient of determination ($R^{2}$) obtained from SigmaPlot software and the adjustable coefficient ($r_{adj}^{2}$) calculated from Equation (5) [35]:

(5) $$r_{adj}^{2}=1-\frac{\left(1-R^{2}\right)\left(N-1\right)}{\left(N-m-1\right)}$$

where N is the number of data points, and m is the total number of independent variables. By comparing these two values, the small difference implies that these independent variables have less impact on the dependent variables.

By comparing the correlation values, the pseudo-second-order kinetics had a better fit than all the other models at any carbonization temperature (Table 1). In addition, SMX adsorption was a good fit only with pseudo-second-order kinetics while the ENR was a good fit with the pseudo-first- and -second-order models. The differences in $R^{2}$ and $r_{adj}^{2}$ were quite large (6–33%) for the SMX pseudo-first-order kinetics, while these differences were <0.13% with the pseudo-second-order kinetics, confirming the greater usefulness of all the parameters for ENR adsorption than for SMX (Table 1). This also indicated that the chemisorption was dominant and possibly involved the primitive valence forces from the electron exchange between the antibiotic molecules and adsorbent surface functional groups [36].

**Figure 4:**
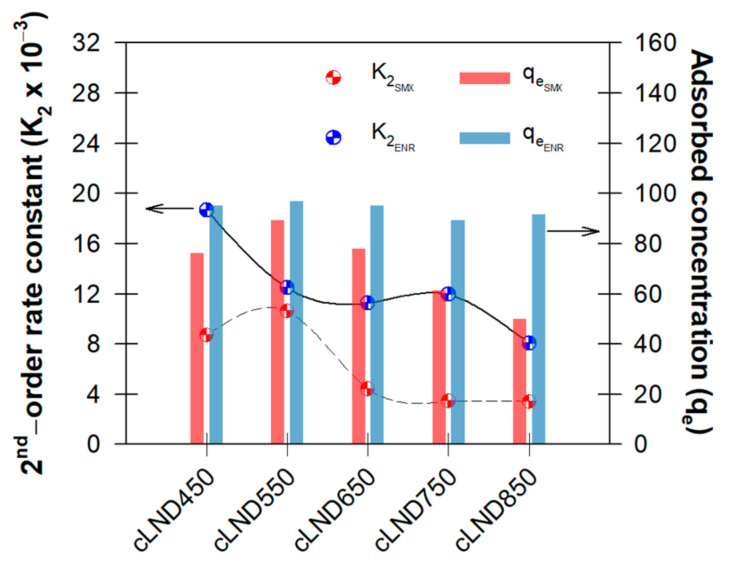
Changes in the pseudo-second-order reaction rates ($K_{2}$) and adsorbed concentrations ($q_{e}$).

Changes in the pseudo-first-order rates ($K_{1}$) and pseudo-second-order rates ($K_{2}$) for ENR adsorption on cLND had the same trend, with both having high values of $r_{adj}^{2}$ for any cLND (0.924–0.998%), corresponding to the good fit of the adsorption data for both models, as discussed earlier (Table 1 and Figure 4 and Figure S1). However, the changes in the reaction rates for SMX adsorption fluctuated more for $K_{1}$ and only had a high $r_{adj}^{2}$ (>0.902%) for the high carbonization temperature (>750 °C), indicating that these temperatures had a high impact on the adsorption behavior of higher hydrophilic molecules under our experimental conditions (Figure S1). As the ENR correlations were close to 1 with all the cLND types, these calculated parameters were reliable and could be used to explain the adsorbate adsorption behaviors in the aqueous phase, confirming the chemisorption process. 

Considering the $K_{2}$ values of SMX adsorption, cLND550 had the highest adsorption rates, confirming that the higher carbonization temperature might not be necessary (Figure 4). However, adsorbent carbonization was still needed to allow cLND to provide interactions between both the hydrophilic functional group (-COOH, -OH, and Si-OH) and the hydrophobic functional group (CH_3_-CH_2_ and C = C), as well as generating numerous pore structures for the better adsorption of organic substances.

All the $q_{e}$ values indicated the cLND had better adsorption for ENR than for SMX (~50–89-mg SMX g^−1^-cLND versus ~89–97-mg ENR g^−1^-cLND), indicating that cLND was a suitable adsorbent for the hydrophobic compound at any carbonization temperature (Table 1). Notably, for ENR, an adsorbed amount almost showed complete adsorption (100 mg g^−1^) at the lower carbonization temperature (Figure 4), because ENR was prone to being adsorbed at a lower carbonization temperature. With the SMX, $q_{e}$ was the highest with cLND550 (~89 mg g^−1^) in the pseudo-second-order rates and continued to decrease with higher carbonization temperatures, which corresponded to the highest kinetic order rates (Figure 4). 

The difference between $q_{e}$ obtained from the experiments ($q_{exp}$) and that obtained from the calculated equations ($q_{cal}$) could also explain the best fit model. The results showed that all $q_{exp}$ values were higher than the ones obtained from the equations (Table 1). The differences were larger for SMX adsorption, especially for the pseudo-first-order kinetic models (6–20%); however, this was much smaller for ENR adsorption for both models (<1.6%). These calculations supported using both models to explain the ENR adsorption mechanism.

The results from fitting the Elovich model produced a bet fit for SMX adsorption ($r_{adj}^{2}$ = 0.938–0.991%) compared to ENR adsorption ($r_{adj}^{2}$ = 0.403–0.616%) (Table 1). Although the suitably fit with the Elovich model lends credence to the adsorbent having a heterogeneous surface, the adsorbed amount variation between sampling points could lead to an incorrect evaluation of the adsorbent surface. This could be further explained by the rapid ENR adsorption in the early stage, which approached >70% after 4 h of sampling, regardless of the carbonization temperature, thus resulting in a smaller adsorption variation afterward (ln T > 0.7). However, the adsorption surface cannot be entirely ruled out for homogeneity, as the SEM images showed its nonuniformity for the selected two carbonization temperatures (Figure 1). 

Both Elovich parameters (a and b) for the antibiotic adsorption varied with the SMX adsorption more than for the ENR (Figure S2). The a and b values were high at the lower carbonization temperature (450 °C) and continued to decrease when the carbonization temperature increased. Notably, the Elovich parameter (a) can vary extremely (from 4.4 × 10^18^ to 1.52 mg g^−1^min^−1^) based on the calculated changes in the approaching equilibrium parameters (R_E_) [37].

**Figure 5:**
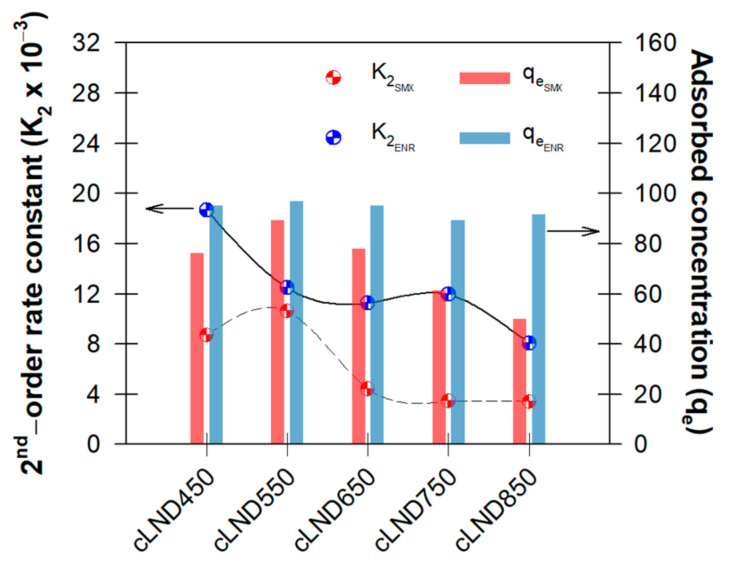
Intraparticle diffusion mechanism plots with varying cLND for (**A**) SMX or (**B**) ENR.

We used the equation model modified from Weber and Morris to determine the adsorption behavior between antibiotics and active sites and whether intraparticle diffusion governed the overall adsorption process [38,39]. The results showed that none of the y-intercepts (C) for the linear intraparticle diffusion equations (Equation (4)) were zero. However, SMX and ENR adsorption produced different patterns that resulted in single-linearity characterization for SMX and double-linearity characterization for the ENR (Figure 5). These stages represented the diffusion type order (external followed by internal diffusion). 

The results for SMX adsorption showed that more than one stage possibly occurred for cLND with a carbonization temperature <650 °C (Figure 5A). The $R^{2}$ was high (>0.93) at all carbonization temperatures (Table 1 and Figure 5A). Here, the diffusion behavior may have occurred simultaneously once the adsorbent met the antibiotic molecules; however, with this relatively high initial concentration, instant adsorption may have occurred before our first sampling (2 h). Therefore, instant adsorption can be easily overshadowed, and consequently, ruling out intraparticle diffusion as the rate-limiting step might be inexact.

For ENR adsorption, the fitted curves showed two distinctive slopes (i.e., K_d1_ and K_d2_) divided at the second sampling (t^1/2^ = 1.414), followed by gradually smaller adsorption variations between the samples until the end of the runs for all carbonization temperatures (Figure 5B). This rapid change in the adsorption rates indicated instant adsorption on the adsorbent surface and that the following slower adsorption rates indicated diffusional phenomenon inside the adsorbent particles [40]. However, the low $R^{2}$ values (<0.85%) and a larger difference between $R^{2}$ and $r_{adj}^{2}$ indicated that this model may not be suitable to explain the adsorbent diffusion mechanisms and also implied that there were other diffusional phenomena controlling the adsorption rates.

### 3.3. Adsorption Isotherms

The aim for the adsorption isotherm analysis between the cLND and antibiotics is to evaluate their adsorption affinity and describe the equilibrium relationships between them. Adsorption isotherm determination is of importance, as it can provide supported data for designing an efficient adsorption system and for improving the adsorption pathways. In this study, we used five isotherm models to characterize the antibiotic molecular distribution at varying equilibrium concentrations obtained from the equilibrium adsorption experiment. The five models used in this study were: Freundlich, Langmuir (linear form Type 1), Langmuir (linear form Type 2), Temkin, Dubinin-Radushkevich (D-R), and Jovanovic (Equations (6)–(16), respectively) [35,41,42,43].

(6) Freundlich isotherm: qe=KfCe1n

(7) Linearized Freundlich equation: lnqe=lnKf+1nlnCe

(8) $$\mathrm{Langmuir}\;\mathrm{isotherm}:\;q_{e}=\frac{q_{m}\cdot b\cdot C_{e}}{1+b\cdot C_{e}}$$

(9) $$\mathrm{Linearized}\;\mathrm{Langmuir}\;\mathrm{Type}\;1\;\mathrm{equation}:\;\frac{1}{q_{e}}=\left[\frac{1}{q_{m}b}\right]\frac{1}{C_{e}}+\frac{1}{q_{m}}$$

(10) $$\mathrm{Linearized}\;\mathrm{Langmuir}\;\mathrm{Type}\;2\;\mathrm{equation}:\;\frac{C_{e}}{q_{e}}=\left[\frac{1}{q_{m}}\right]C_{e}+\frac{1}{q_{m}b}\;$$

(11) $$\mathrm{Temkin}\;\mathrm{isotherm}:\;q_{e}=\left(\frac{RT}{b_{T}}\right)\ln\left(A_{T}C_{e}\right)\;$$

(12) $$\mathrm{Linearized}\;\mathrm{Temkin}\;\mathrm{equation}:\;q_{e}=\left(\frac{RT}{b_{T}}\right)\ln A_{T}+\left(\frac{RT}{b_{T}}\right)\ln C_{e}$$

(13) $$\mathrm{Dubinin}-\mathrm{Radushkevich}\;(D-R):\;q_{e}=Q_{m}e^{-\left(B\varepsilon^{2}\right)};\;\varepsilon =RT\ln\left(1+\frac{1}{C_{e}}\right)\;$$

(14) $$\mathrm{Linearized}\;\mathrm{Dubinin}-\mathrm{Radushkevich}\;\mathrm{equation}:\;\ln q_{e}=\ln Q_{m}-B\varepsilon^{2}$$

(15) $$\mathrm{Jovanovic}\;\mathrm{isotherm}:\;q_{e}=\frac{q_{m}}{e^{K_{j}C_{e}}}$$

(16) $$\mathrm{Linearized}\;\mathrm{Jovanovich}\;\mathrm{equation}:\;\ln\;q_{e}=\ln q_{m}-K_{j}C_{e}$$

where $C_{e}$ (mg L^−1^) is the antibiotic equilibrium concentration, $q_{e}$(mg g^−1^) is the cLND adsorption capacity at the equilibrium, $q_{m}$ (mg g^−1^) is the cLND theoretical maximum adsorption capacities, b (L mg^−1^) is the Langmuir energy constant related to the adsorption heat, $K_{f}$ (mg g^−1^(L mg^−1^)^1/n^) is the Freundlich constant, 1n is the Freundlich adsorption intensity, R (8.314 J mol^−1^K^−1^) is the universal gas constant, T (K) is the temperature, $b_{T}$ (J mol^−1^) is the Temkin constant, $A_{T}$ (L mol^−1^) is the Temkin Equilibrium binding constant, $Q_{m}$ is the adsorption saturation capacity, B is a Dubinin-Radushkevich constant, and $K_{j}$ is the Jovanovich constant. Again, we used $R^{2}$ and $r_{adj}^{2}$ to evaluate the best fit isotherm model. 

We selected cLND550 to evaluate further adsorption mechanisms, because the adsorption kinetic experiments indicated that it was a better adsorbent for two different antibiotic families. Both antibiotics were again used in this experiment, because their different chemical properties may have affected the adsorption results. In addition, we selected cLND450 to compare the adsorption behavior with the cLND550 results as, at this carbonization temperature, some hydrophilic functional groups still existed. 

Based on the $R^{2}$ and $r_{adj}^{2}$_j_ values, the adsorption process followed the order of Langmuir, Temkin, and D-R, indicating that antibiotic molecules formed multilayer coverage on the cLND heterogeneous surface (Table 2). The high $R^{2}$ values indicated that the Langmuir isotherm better described the SMX adsorption with both cLNDs similar to the ENR (Table 2). The Langmuir maximum monolayer adsorption capacity ($q_{m}$) was different between the SMX and ENR. The $q_{mENR,450}$ and $q_{mENR,550}$ values were relatively close (100.01 versus 104.16 mg g^−1^), indicating that ENR was prone to be adsorbed with cLND regardless of the carbonization temperature due to its hydrophobicity. The $q_{mSMX,450}$ and $q_{mSMX,550}$ values differed by 17% (Table 2), indicating that cLND550 was a much better adsorbent for SMX than cLND450, corresponding with the previous experiments that showed a better adsorption for cLND550.

Notably, the Langmuir isotherms can be linearized into four types of equations, depending on the variables plotted for the X- and Y-axis [31]. Selected linearized Langmuir equations were the only ones that had $R^{2}$ values >0.9. As it can be seen, both linearized equations provided a high correlation coefficient, supporting the well-described with Langmuir isotherm. Having said that, it does not necessarily mean all linearized Langmuir equations will always provide best fit. The difference in the linearized *Y*-axis and *X*-axis caused these fit discrepancies. Among them, Langmuir type 2 ($C_{e}/q_{e}$ versus $C_{e}$) generally provide minimal error distributions between sampling points, hence giving a better fit than the others [25]. The Langmuir isotherm was further calculated for the reaction favorability and the isotherm type (Equation (17)).

(17) $$R_{L}=\frac{1}{1+bC_{o}}$$

where $R_{L}$ is a dimensionless equilibrium parameter, and $C_{o}$ (mg L^−1^) is the antibiotic initial concentration. The results showed that all $R_{L}$ values for any cLND adsorption were between 0 and 1 (0.052–0.966), indicating that the adsorption was a favorable process under the conditions applied. These numbers also coincided with the 1/n values (<1 for complete antibiotic adsorption) obtained from the Freundlich isotherm model, which also indicated the favorability of SMX and ENR adsorption onto cLND.

### 3.4. Influential Effect Experiments

#### 3.4.1. Effect of pH

**Figure 6:**
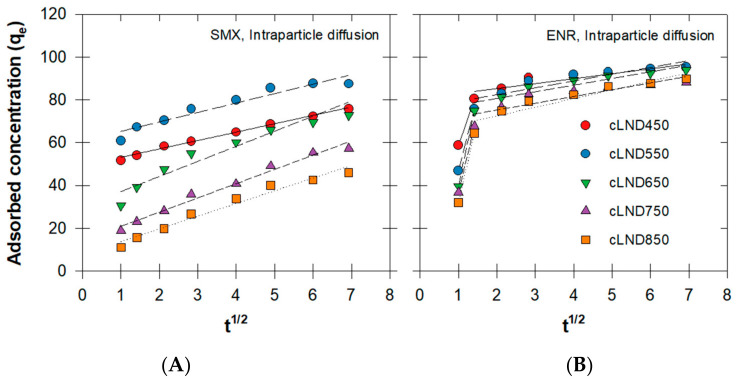
Effect of (**A**) initial pH and (**B**) humic acid concentrations on SMX or ENR removal efficiency using cLND550.

**Figure 7:**
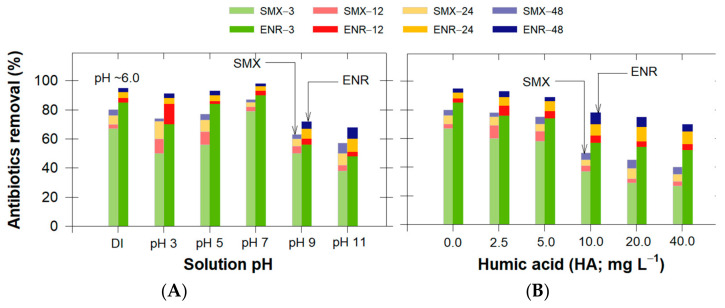
Effect of (**A**) bicarbonate and (**B**) chloride concentrations on SMX or ENR removal efficiency using cLND550.

Typical environmental parameters that could potentially affect the adsorption efficiency were investigated using cLND550 against the presence of SMX or ENR. The parameters considered were: solution pH, organic matter, represented by humic acids (HA), bicarbonate (HCO_3_^−^), and chloride (Cl^−^). We reported the changes in antibiotic removal efficiency at different timelines (3, 12, 24, and 48 h) to demonstrate how these effects influenced their adsorption mechanisms (Figure 6 and Figure 7).

Although the typical antibiotic-containing discharge water from animal farming is slightly alkaline, the pH from typical wastewater treatment plants can range between 2 and 10. The results showed that the adsorption efficiency decreased for alkaline conditions (Figure 6A). The adsorbate–adsorbent equilibrium was reached at approximately 24 h, as only slight changes were observed at 48 h. Compared with the control (adsorption in DI; pH~6.0), the SMX adsorption efficiency decreased up to 23% at pH 11, while that of ENR decreased up to 27%, indicating that the antibiotic adsorption was pH-dependent (Figure 6A). This also depended on the antibiotic chemical characteristics, as an antibiotic can be present in cationic, anionic, and neutral forms under different pH conditions. Since the important key role in the adsorption process was based on the catalyst surface, we determined the cLND550 point of zero charges (pH_pzc_) was approximately 8.5. When the experimental pH > pH_pzc_, the cLND550 surface became negatively charged, favoring cationic species adsorption and vice versa; when the experimental pH < pH_pzc_, the positively charged cLND550 surface favored anionic species adsorption.

SMX and ENR had two pKa (1.85 and 5.29 for SMX and 6.19 and 7.91 for ENR) [44,45]. For pH < pKa1, the antibiotic forms were positively charged (SMX^+^ and ENR^+^), while, for pH > pKa2, the antibiotic forms were negatively charged (SMX^−^ and ENR^−^). Therefore, the antibiotic zwitterionic forms (SMX^+/−^ and ENR^+/−^) were present when the pH was between pKa1 and pKa2. 

The antibiotic rapid adsorption in the first stage (3 h) was due to these opposite charges between the adsorbate and adsorbent. As discussed earlier in the adsorption kinetic experiment, the adsorption efficiency between ENR and SMX was substantially different (Figure 3). Notably, ENR adsorption on cLND550 was high (~91%), even though both ENR and cLND550 had a positive charge. This can be explained by the ENR’s low water solubility, facilitating the hydrophobic adsorption and the π–π interaction between the ENR and aliphatic functional groups on cLND550 (Figure 2) [46,47,48]. In addition, the ENR adsorption could have been due to the H bonding occurring between fluorine atoms in the ENR structure and -OH functional groups on the cLND surface (Figure 2) [49]. These were possibly the main reasons for the better ENR adsorption under acidic conditions despite both ENR and cLND550 having a positive charge. 

Since ENR has a lower water solubility than SMX (~146 mg L^−1^ versus 281 mg L^−1^), SMX hydrophobic adsorption and the π–π interaction could be less, resulting in a lower adsorption efficiency than for ENR [50]. In addition, under acidic conditions, the SMX water solubility was much lower than for alkaline conditions, facilitating a greater adsorption for SMX with this pH [51]. 

When 3 < pH < 6, the same ENR adsorption behavior was expected, as discussed earlier. By increasing the pH from 5 to 7, the SMX adsorption efficiency increased from 77 to 87% (Figure 6A), mainly due to the electrostatic attraction occurring from the increased SMX charge distribution toward SMX^−^ from the SMX deprotonation, making it more prone to being adsorbed on the cLND550 positively charged surface (cLND^+^). At pH 7, the ENR adsorption reached its maximum adsorption capacity of 98% (Figure 6A). Here, the hydrophobic interaction, π–π interaction, and H bonding were more pronounced with aromatic-containing molecules -OH and Si-OH on the cLND550 surface (Figure 2) [47]. A similar observation for a neutral pH was reported for ENR adsorption on highly pyrolytic corn stalk materials [52].

For strong alkaline conditions (pH 9–11), both antibiotics had negative charges, increasing the electrostatic repulsion with cLND^−^ [53,54]. Therefore, the SMX removal efficiencies decreased to 63% and 57% for pH 9 and 11, respectively, while those of ENR decreased to 72% and 68%, respectively (Figure 6A). The ongoing adsorption for this pH range was observed, which could be attributed to the interaction between the antibiotic molecule and the existing cLND550 functional groups, as discussed earlier. 

#### 3.4.2. Effect of Humic Acids (HAs)

Since real antibiotic-containing discharge water usually contains natural organic matter, we investigated this influential effect on the antibiotic adsorption efficiency by varying the HA content between 2.5 mg L^−1^ and 40 mg L^−1^. Compared to the control (no HA), the results showed that HA potentially suppressed the removal efficiency by up to 40% for SMX and by up to 25% for ENR (Figure 6B). 

HA is usually rich in numerous functional groups embedded on the particle surface of the HA, such as carboxylic acid and aromatic and phenolic groups [55]. This functional group existence enabled stronger interactions with other foreign chemicals on the cLND surface via either H bonding or π–π interactions. Among these, carboxylic acid is naturally deprotonated, making the HA negatively charged for most environmental pH ranges. Once presented together with cLND under normal pH conditions, cLND^+^ (pH_pzc_ = 8.5) spontaneously became available at adsorptive sites that favored HA adsorption, giving fewer sites for antibiotic adsorption. In addition, the existence of nonpolar functional groups can further adsorb HA, which can potentially compete with target antibiotic contaminants with a higher HA content. Hou et al. [10] showed that HA possibly combined with the available antibiotics and formed a more soluble complex compound that can potentially minimize the adsorption performance. However, antibiotic adsorption still occurred, indicating that the cLND surface could still provide unbound functional groups with antibiotic molecules. The better adsorption by ENR than SMX could have been due to the electrostatic attraction between ENR^+^ and HA^−^ for these normal pH conditions. Although most suspended solids would be removed during the preliminary process in the WWTP, other organic constituents may be of concern. This information suggested other means to initially separate abnormally high humic colloids or other organic-containing contents from the water before it entered the adsorption unit. 

#### 3.4.3. Effect of Anionic Constituents

Since bicarbonate (HCO_3_^−^) and chloride (Cl^−^) are some of the most prominent anionic compounds in nature, we individually varied these two anionic concentrations up to 800 mg L^−1^ for neutral pH conditions. The results showed that the increase in these two ions slightly decreased the antibiotic adsorption efficiency (Figure 7). HCO_3_^−^ had fewer negative effects on antibiotic adsorption than Cl^−^. This can be explained by the increase in the anionic concentration simultaneously filling the aqueous solution with anionic molecules that later underwent electrostatic attraction with cLND^+^, consequently blocking the sorption sites for antibiotic adsorption. 

Other than competing with the sites with cLND^+^, the interference was more pronounced with SMX adsorption. Since this reaction occurred under neutral pH conditions, the repulsion interaction between anionic ions and SMX^−^ also occurred, suppressing its electrostatic attraction with cLND^+^. This anionic existence in the adsorption process may not be problematic due to the lower magnitude of interference. While our highest tested concentration was far less than the natural possible conditions (Cl^−^ ~19,800 mg L^−1^ for seawater; [56]), the ENR-adsorbed concentrations on bamboo biochar in the presence of 3000 mg L^−1^ Cl^−^ were reduced by only 25% [57]. Overall, the results confirmed that the negative influence of anions on the SMX/ENR adsorption should not be neglected at high anionic concentrations (>200 mg·L^−1^).

### 3.5. Bacterial Activity

**Figure 8:**
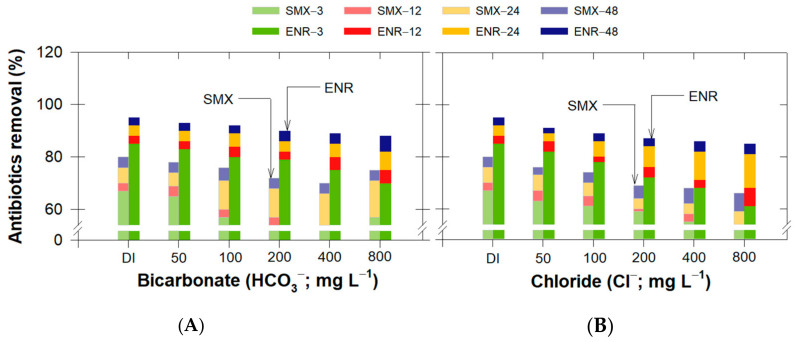
(**A**) Clear zone difference of *Staphylococcus aureus* ATCC 25923 after 24-h adsorption (C_Δ-24h_) of ENR onto each type of cLND (cLND450 and cLND550), and (**B**,**C**) clear zone examples at two ENR concentrations (20 and 40 mg L^−1^) onto cLND550.

As stated earlier in the adsorption kinetics section, chemisorption plays a dominant role during the adsorption process, and so, the desorption processes can be ignored. To validate this statement, we used a growth inhibition zone experiment and testing Gram-negative *Staphylococcus aureus* (ATCC 25923) bacteria with treated water (filtrated water) and cLND (retentate after filtration). We selected only ENR as the target representative, as it was more frequently detected [58], and it has greater absorptivity on cLND at any carbonization temperature. The clear zone difference percentage after 24 h of adsorption (C_Δ-24h_) was calculated and plotted against the ENR initial concentration (Figure 8A).

Using cLND at 300 mg, ~100% of C_Δ-24h_ at 20 mg L^−1^ ENR was observed, indicating that there was no clear zone after cLND adsorption, and thus, the cLND completely removed the ENR (Figure 8B). Conversely, at the lower cLND amount (30 mg), the ENR residue was still in the solution, resulting in ~7–~11% C_Δ-24h_ (Figure 8A). As such, regardless of the cLND type or amount, the increase in the ENR initial concentration substantially increased C_Δ-24h_ (Figure 8B,C). This can be explained by the reduced availability of the adsorptive sites on the cLND surface to adsorb ENR, as the available ENR molecules had already been chemisorbed on the surface. The cLND550 C_Δ-24h_ was slightly higher than for cLND450, signifying that the cLND550 could better adsorb antibiotics than cLND450 (Figure 8A). 

Banana peel biochar can serve as a growth inhibitor for *E. coli*, because the natural potassium chloride in the biochar can hinder the bacterial cellular activities [59]. Our cLND had no such effect on the *S. aureus* ATCC 25923 strains, indicating that the clear zone occurrence was solely from the existing adsorbed ENR, not from the cLND itself, and that any cLND leached into the environment would not interfere with any local organisms. 

**Figure 9:**
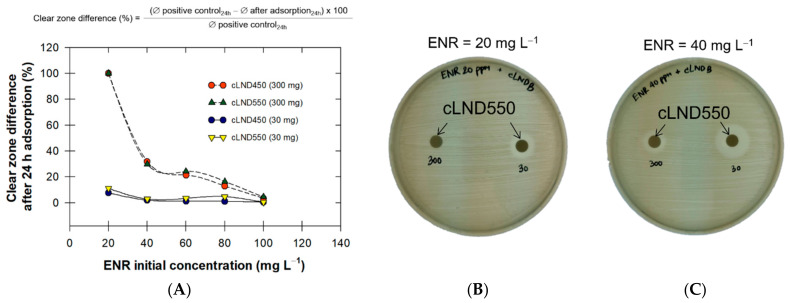
Changes in the clear zone after day 3 (C_Δ-3d_) of *Staphylococcus aureus* ATCC 25923 following ENR adsorption onto cLNDs.

A key to being a compatible adsorbent for real applications is that the adsorbent should retain the adsorbate without releasing it into the aqueous solution. Again, we used this sensitive bacterial growth inhibition experiment on the retentate cLND obtained from both the cLND adsorption experiments (cLND450 and cLND550) with various initial ENR concentrations. A comparison was made between day 1 and day 3 and presented as an increase in *S. aureus* growth (change percentages in the clear zone, C_Δ-3d_) at different ENR initial concentrations (Figure 9).

With the different cLND amounts or different carbonization temperatures, both cLNDs had the same clear zone trend, where an increase in the ENR initial concentration increased the C_Δ-3d_. Although C_Δ-3d_ was the highest at 100 mg L^−1^ ENR concentration in any of the experimental setups, the C_Δ-3d_ value was ~9.6% (Figure 9). This indicated that the cLND was still beneficial, as it retained (less desorption) most of the adsorbed ENR. 

The cLND550 retained better microbial activity than the cLND450 at any ENR initial concentration based on the value of C_Δ-3d_ (2.35% for 30 mg cLND and 1.01% for 300 mg cLND), as shown in Figure 9. The ENR stronghold on cLND can be explained by both physical adsorption and chemisorption, with more than one interaction potentially occurring, such as the π–π interaction, hydrophobic adsorption, H bonding, and pore filling between antibiotics on the biochar surface [60]. The unchanged C_Δ-3d_ value for cLND450 at 30 mg indicated little microbial activity could be expected that might interfere with the cLND adsorption activity under adsorptive competition between adsorbates. Notably, we used a much higher ENR concentration in this experiment compared to the frequently detected concentration, thus confirming that cLND could efficiently adsorb ENR even at exceptionally high concentrations, which could compromise the effectiveness of the other adsorbate organic constituents.

## 4. Conclusions

Carbonized leonardite (cLND) was successfully prepared from a byproduct generated from lignite coal that was carbonized at a constant heat rate to the desired temperatures between 450 °C and 850 °C under a N_2_ flow stream. Of these carbonization temperatures, the cLND550 product had the highest adsorption capability for SMX and ENR, because the cLND550 had both hydrophobic and hydrophilic active functional groups on the cLND surface. The nonlinear kinetics fitting results showed that the pseudo-second-order kinetics model was more suitable for describing the cLND and SMX or ENR adsorption. Among the several isotherm models, both the SMX and ENR adsorption equilibrium data fitted well with Langmuir isotherms. The cLND yielded maximum adsorption capacities of 104.167 mg g^−1^ (ENR) and 45.249 mg g^−1^ (SMX). For neutral pH conditions, antibiotic adsorption revealed the highest removal efficiency due to the electrostatic interaction and H bonding between the antibiotic and cLND. Anionic and organic constituents suppressed the antibiotic adsorption, mostly resulting from the charge attraction between cLND and these ions, which competed with the available antibiotics. Microbial activities confirmed that cLND450 and cLND550 successfully adsorbed ENR at varying ENR initial concentrations, and the percentage desorption difference after 3 d was relatively low (1.70–35%). Overall, this work showed the potential good utilization of low-ranked coals to efficiently adsorb emerging contaminants, such as antibiotics, and the study also provided proof that cLND was a suitable adsorbent for antibiotics and could be applied to combat various kinds of water pollutants.

## Figures and Tables

**Table 1:** Kinetic parameters for SMX and ENR adsorption onto varying types of cLND.

Kinetic Model Parameters		Sulfamethoxazole (SMX)					Enrofloxacin (ENR)				
		cLND 450	cLND 550	cLND 650	cLND 750	cLND 850	cLND 450	cLND 550	cLND 650	cLND 750	cLND 850
$\mathrm{Experimental}\;q_{e}$											
$q_{\exp}$	mg g^−1^	75.812	87.603	75.519	47.832	45.992	94.939	95.431	93.710	88.447	89.107
**First-order kinetic model**											
$q_{cal}$	mg g^−1^	71.331	80.543	62.940	43.376	40.399	94.441	95.149	92.166	88.304	89.497
$K_{1}$	h^−1^	0.737	0.893	0.161	0.847	0.086	0.916	0.681	0.637	0.604	0.498
$R^{2}$	-	0.820	0.906	0.880	0.957	0.961	0.987	0.982	0.974	0.983	0.967
$r_{adj}^{2}$	-	0.618	0.791	0.737	0.902	0.911	0.970	0.958	0.940	0.961	0.924
**Second-order kinetic model**											
$q_{cal}$	mg g^−1^	76.336	89.286	78.125	61.38	50.000	95.238	97.087	95.238	89.286	91.043
$K_{2}$	g mg^−1^h^−1^	8.7 × 10^−3^	10.6 × 10^−3^	4.4 × 10^−3^	3.4 × 10^−3^	3.4 × 10^−3^	18.7 × 10^−3^	12.5 × 10^−3^	11.2 × 10^−3^	11.9 × 10^−3^	8.1 × 10^−3^
$R^{2}$	-	0.997	0.999	0.995	0.994	0.995	0.999	0.999	0.999	0.999	0.999
$r_{adj}^{2}$	-	0.993	0.998	0.988	0.986	0.988	0.998	0.998	0.998	0.998	0.998
**Elovich model**											
a	mg g^−1^h^−1^	21,552	39,988	161.8	22.1	23.9	57,835	3526.7	1756.9	1342.5	568.5
b	g mg^−1^	0.163	0.141	0.088	0.099	0.107	0.129	0.096	0.090	0.092	0.081
$R^{2}$	-	0.973	0.983	0.996	0.991	0.980	0.775	0.794	0.754	0.699	0.819
$r_{adj}^{2}$	-	0.938	0.961	0.991	0.979	0.954	0.534	0.569	0.497	0.403	0.616
**Intraparticle diffusion**											
$K_{d1}$	mg g^−1^h^−1/2^	3.891	4.391	7.109	6.648	5.850	54.463	70.132	83.980	70.993	78.532
$C_{1}$	mg g^−1^	49.344	60.910	29.834	5.979	8.287	3.551	23.186	43.631	19.242	46.47
$K_{d2}$	mg g^−1^min^−1^	-	-	-	-	-	1.641	2.367	2.439	1.067	3.011
$C_{2}$	mg g^−1^	-	-	-	-	-	84.219	80.520	78.110	91.257	69.972
$R^{2}$	-	0.988	0.932	0.949	0.967	0.977	0.815	0.817	0.802	0.627	0.822
$r_{adj}^{2}$	-	0.972	0.847	0.884	0.924	0.947	0.608	0.612	0.584	0.292	0.622

**Table 2:** SMX or ENR kinetic parameter comparisons for two types of cLND (cLND450 and cLND550).

Adsorption Isotherm Parameters		Sulfamethoxazole (SMX)		Enrofloxacin (ENR)	
		cLND 450	cLND 550	cLND 450	cLND 550
Freundlich isotherm					
$K_{f}$	mg g^−1^(L mg^−1^)^1/n^	11.987	16.211	25.554	18.460
1n	-	0.394	0.330	0.423	0.512
$R^{2}$	-	0.822	0.844	0.818	0.877
$r_{adj}^{2}$	-	0.622	0.664	0.614	0.731
**Langmuir isotherm** **(Linearized Langmuir Type 1 equation)**					
$q_{m}$	mg g^−1^	46.083	50.761	120.482	129.870
b	L mg^−1^	0.179	0.239	0.014	0.094
$R^{2}$	-	0.951	0.958	0.934	0.953
$r_{adj}^{2}$	-	0.888	0.904	0.851	0.893
$R_{L}$	-	0.101–0.691	0.077–0.626	0.588–0.966	0.175–0.810
**Langmuir isotherm** **(Linearized Langmuir Type 2 equation)**					
$q_{m}$	mg g^−1^	38.610	45.249	100.003	104.167
$b_{T}$	L mg^−1^	0.309	0.366	0.229	0.147
$R^{2}$	-	0.974	0.985	0.956	0.946
$r_{adj}^{2}$	-	0.940	0.965	0.900	0.877
$R_{L}$	-	0.061–0.564	0.052–0.522	0.080–0.636	0.120–0.731
**Temkin isotherm**					
b	J mol^−1^	275.893	251.898	101.803	95.889
$A_{T}$	L mol^−1^	2.511	3.573	1.717	1.113
$R^{2}$	-	0.847	0.866	0.836	0.911
$r_{adj}^{2}$	-	0.670	0.708	0.649	0.802
**Dubinin-Radushkevich isotherm**					
$Q_{m}$	mg g^−1^	13.330	14.013	14.879	14.441
B	-	2.92 × 10^−6^	3.53 × 10^−6^	4.09 × 10^−6^	−4.17 × 10^−6^
$R^{2}$	-	0.831	0.847	0.872	0.854
$r_{adj}^{2}$	-	0.639	0.670	0.720	0.684
**Jovanovich isotherm**					
$q_{m}$	mg g^−1^	17.764	23.729	42.636	32.858
$K_{j}$	-	0.041	0.033	0.041	0.052
$R^{2}$	-	0.596	0.639	0.62	0.724
$r_{adj}^{2}$	-	0.248	0.310	0.292	0.445

## Data Availability

The authors confirm that the data supporting the findings of this study are available within the article.

## Supplementary Materials

The following supporting information can be downloaded at https://www.mdpi.com/article/10.3390/antibiotics11091261/s1: Figure S1: Changes in the pseudo-first-order reaction rates ($K_{1}$) and adsorbed concentrations ($q_{e}$) following varying types of cLND adsorption. Figure S2: Changes in the Elovich parameters (a and b) following varying types of cLND adsorption.
