# Supplementary material for: Enrofloxacin and Sulfamethoxazole Sorption on Carbonized Leonardite: Kinetics, Isotherms, Influential Effects, and Antibacterial Activity toward S. aureus ATCC 25923
Source: Antibiotics (Basel). 2022 Sep 16;11(9):1261. doi: 10.3390/antibiotics11091261 (PMC9495318; doi:10.3390/antibiotics11091261)
Supplement: Supplementary file 1 [file antibiotics-11-01261-s001.zip › antibiotics-1850691-supplementary.pdf]

Supplementary Materials

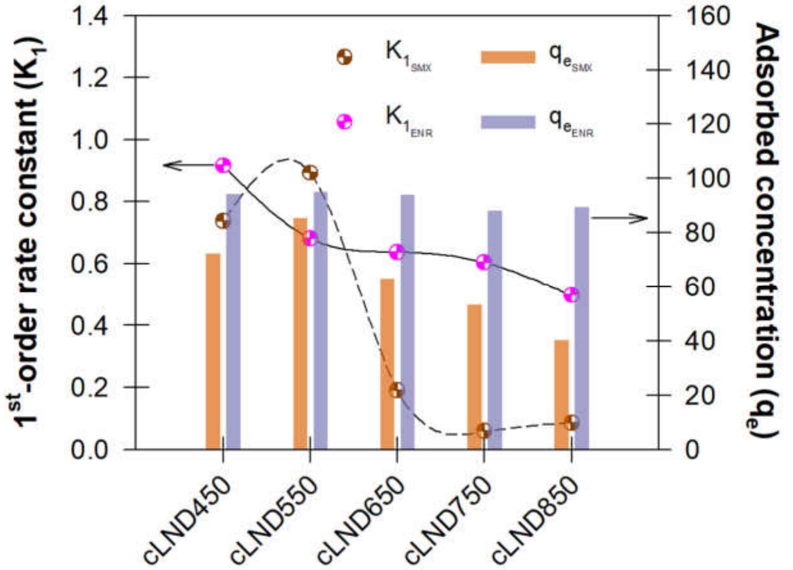

Figure S1. Changes in pseudo-1st order reaction rates (K_1) and adsorbed concentrations (q_e) following varying types of cLND adsorption.

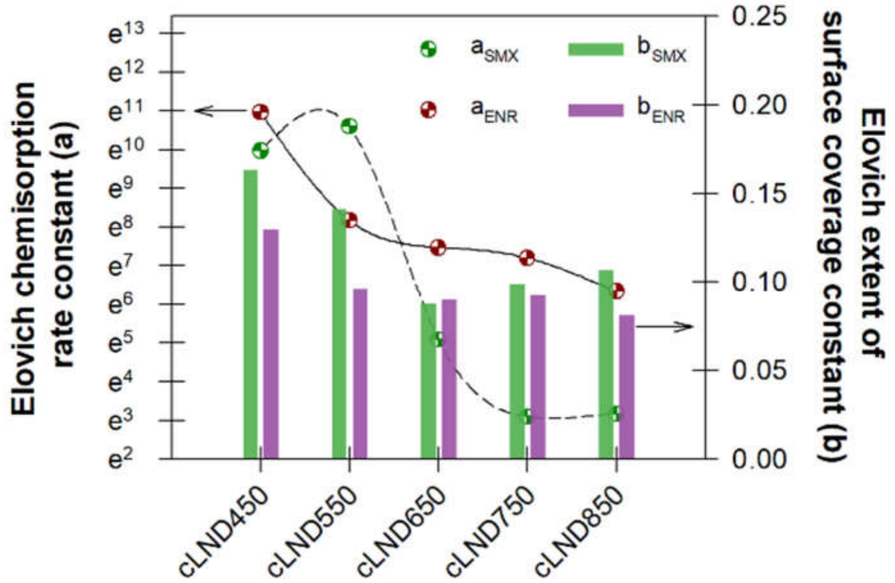

Figure S2. Changes in the Elovich parameters (a, b) following varying types of cLND adsorption.
